# Fatty acid-binding protein 2 in inflammatory bowel disease: mechanistic insights and translational perspectives

**DOI:** 10.3389/fmed.2026.1901900

**Published:** 2026-08-14

**Authors:** Shuhang Zhong, Kaishuo Lv, Xiaoqing Liu, Junru Zhou, Jiayu Ye, Zhiyu Yang

**Affiliations:** 1 Department of Gastroenterology and Hepatology, People's Hospital of Jiaozuo City, Jiaozuo, Henan, China; 2 Department of Gastroenterology and Hepatology, Henan Provincial People's Hospital, People's Hospital of Zhengzhou University, Zhengzhou, Henan, China; 3 Department of Laboratory Medicine, Henan Provincial People's Hospital, People's Hospital of Zhengzhou University, Zhengzhou, Henan, China; 4 Department of Gastroenterology and Hepatology, Stanford University School of Medicine, Palo Alto, CA, United States

**Keywords:** biomarker, fatty acid-binding protein 2, inflammatory bowel disease, molecular mechanisms, therapeutic target

## Abstract

Fatty acid-binding protein 2 (FABP2) is an intestine-specific cytoplasmic lipid chaperone with a molecular weight of approximately 14–15 kDa that plays a pivotal role in the uptake, intracellular trafficking, and metabolism of long-chain fatty acids. Emerging evidence indicates that FABP2 is not only a sensitive biomarker of intestinal mucosal injury but also may contribute to the pathogenesis and progression of IBD, potentially through the modulation of PPARγ signaling pathway, although the underlying mechanisms require further investigation. This review systematically summarizes the structural and functional characteristics of the fatty acid-binding protein (FABP) family members, with particular emphasis on the biological features of FABP2. We discuss the involvement of FABP2 in multisystem diseases and highlight its specific relevance in IBD. Furthermore, we summarize current evidence regarding the potential molecular mechanisms by which FABP2 may influence IBD, including its possible roles in intestinal barrier dysfunction, lipid metabolism, gut microbiota-host interactions, and its potential contribution to the attenuation of intestinal inflammation, possibly through regulation proliferation and differentiation of intestinal stem cells. Although the biological investigations of FABP2 in inflammatory bowel disease (IBD) have yielded promising findings, its clinical translation is still confronted with challenges such as the precise assessment of disease activity and the development of safe and effective targeted therapeutics. Collectively, this review provides a comprehensive reference for researchers and clinicians in the IBD field, highlighting the well-supported role of FABP2 as a diagnostic biomarker while discussing its potential as a therapeutic target, an area that warrants further mechanistic and clinical investigation.

## 1 Introduction

Inflammatory bowel disease (IBD) comprises a group of chronic, relapsing inflammatory disorders of the gastrointestinal tract, primarily including ulcerative colitis (UC) and Crohn's disease (CD). The pathogenesis of IBD is multifactorial and is generally attributed to dysregulated immune responses triggered by environmental factors in genetically susceptible individuals (1). The global incidence and prevalence of IBD have risen steadily in recent decades, leading to substantial morbidity, impaired quality of life, and increasing healthcare burdens (2).

Accumulating evidence has highlighted the pivotal roles of intestinal lipid metabolism, epithelial barrier integrity, and gut microbiota–host interactions in IBD pathogenesis (1, 3–5). Among the molecules implicated in these processes, fatty acid-binding protein 2 (FABP2) has gained increasing attention. As a member of the fatty acid-binding protein (FABP) family, FABP2 is an intestine-enriched intracellular lipid chaperone that mediates the intracellular trafficking of long-chain fatty acids in small intestinal epithelial cells and participates in lipid-mediated signal transduction and gene regulation (6). Evidence linking FABP2 to IBD and intestinal inflammation derives from several distinct sources, including human clinical studies, animal models, *in vitro* epithelial or immune-cell experiments, and mechanistic hypotheses based on lipid metabolism and nuclear receptor signaling. Human studies mainly provide associative evidence regarding FABP2 expression, genetic variation, circulating or fecal FABP2 levels, and disease activity or intestinal injury. Animal models offer experimental support for the involvement of lipid metabolism and epithelial barrier dysfunction in intestinal inflammation, but their findings require cautious interpretation when extrapolated to human IBD. *In vitro* studies help clarify potential cellular mechanisms, including fatty acid transport, epithelial barrier regulation, and inflammatory signaling, whereas some proposed pathways remain hypothetical and have not yet been directly validated in IBD patients.

In this context, FABP2 has been proposed to facilitate the delivery of fatty acid ligands to peroxisome proliferator-activated receptors (PPARs), particularly PPARγ, thereby potentially influencing intestinal inflammation and the pathogenesis of IBD; however, this mechanism is mainly supported by experimental and inferential evidence, and the potential involvement of PPARβ/δ in epithelial repair warrants further investigation (7–13). Therefore, throughout this review, we distinguish evidence from human clinical observations, animal studies, *in vitro* experiments, and mechanistic hypotheses where appropriate, in order to clarify the strength, limitations, and translational relevance of current findings.

This review provides a comprehensive overview of the biological characteristics of FABP2, its involvement in IBD and other disease contexts, and its potential clinical applications. A better understanding of the evidence hierarchy and potential mechanistic roles of FABP2 in IBD may facilitate the development of novel diagnostic biomarkers and targeted therapeutic strategies for these challenging disorders.

## 2 Literature search and selection

**Figure 1:**
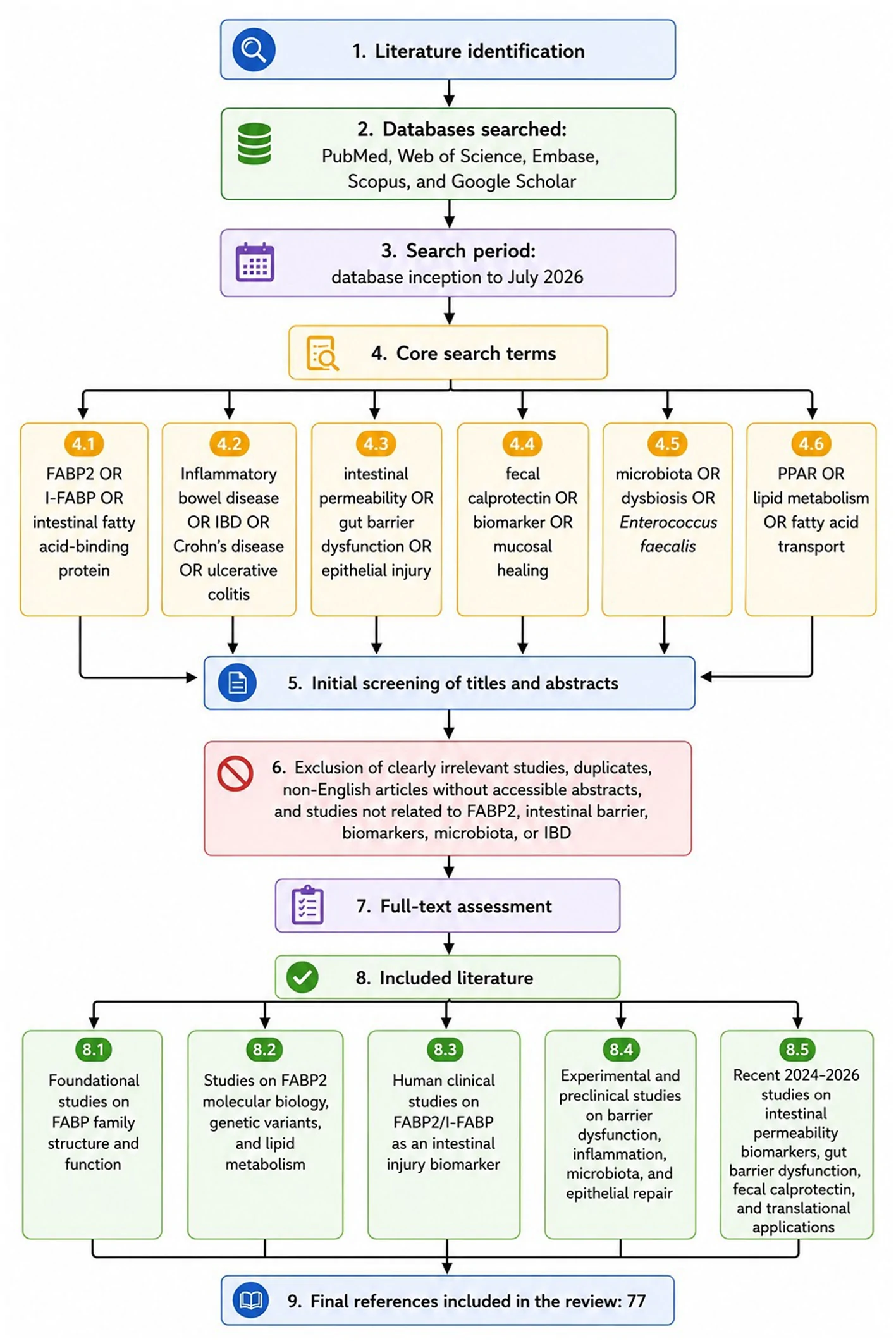
Literature identification and selection process for this narrative review. A structured literature search was performed across major databases to identify studies related to FABP2/I-FABP, intestinal epithelial injury, lipid metabolism, gut barrier dysfunction, microbiota interactions, IBD biomarkers, and translational applications. Because this was a narrative review, the process was used to improve transparency rather than to conduct formal quantitative evidence synthesis.

Because this was a narrative review rather than a formal systematic review or meta-analysis, the literature search was structured but not designed for quantitative evidence synthesis. Relevant literature available up to July 2026 was searched, with particular emphasis on studies published between 2024 and 2026. The overall literature identification and selection process is summarized in Figure 1.

As shown in Figure 1, relevant publications were identified through searches of PubMed, Web of Science, Embase, Scopus, and Google Scholar. Search terms included “FABP2,” “I-FABP,” “intestinal fatty acid-binding protein,” “inflammatory bowel disease,” “Crohn's disease,” “ulcerative colitis,” “intestinal permeability,” “gut barrier dysfunction,” “epithelial injury,” “fecal calprotectin,” “microbiota,” “dysbiosis,” “*Enterococcus faecalis*,” “PPAR,” “lipid metabolism,” and “mucosal healing.” After removing duplicate and clearly irrelevant records, titles and abstracts were screened for relevance. Full texts were then assessed, and studies were included if they addressed FABP family biology, FABP2 structure or function, FABP2-related lipid metabolism, intestinal epithelial injury, IBD biomarkers, gut barrier dysfunction, microbiota interactions, or translational applications relevant to IBD. Foundational older studies were retained when necessary to support established molecular or physiological concepts, while recent studies from 2024 to 2026 were prioritized for sections discussing intestinal permeability biomarkers, gut barrier dysfunction, fecal calprotectin, and emerging translational applications.

## 3 The role of FABP2 in inflammatory bowel disease

### 3.1 The FABP family members and FABP2: structure, function, and tissue distribution

#### 3.1.1 Overview of the FABP family members

Fatty acid-binding proteins (FABPs) are a family of highly conserved, low–molecular weight (14–15 kDa) cytoplasmic proteins that function as intracellular lipid chaperones (14). They reversibly bind long-chain fatty acids and other hydrophobic ligands, thereby facilitating their intracellular trafficking, metabolism, and signaling (15). Multiple tissue-specific isoforms have been identified, each exhibiting distinct expression patterns and specialized biological functions (16).

Major members of the FABP family include liver-type (FABP1), intestinal-type (FABP2), heart-type (FABP3), adipocyte-type (FABP4), and epidermal-type (FABP5) isoforms. These proteins display characteristic tissue distributions and participate in diverse metabolic and regulatory processes, as summarized in Table 1. For instance, FABP1 is predominantly expressed in hepatocytes and renal proximal tubular cells, contributing to hepatic fatty acid uptake and oxidation (14). FABP3 is highly expressed in cardiac and skeletal muscle, where it supports fatty acid–dependent energy metabolism, and FABP4 is abundant in adipocytes and macrophages, modulating lipid storage and inflammatory responses (17).

**Table 1:** FABP family members: tissue distribution and core biological functions.

Family members	Tissue distribution	Core biological functions
Liver-type (FABP1)	Hepatocytes, renal proximal tubular cells (74)	Mediates hepatic fatty acid uptake and oxidation, and participates in the regulation of lipid metabolism (14)
Intestinal-type (FABP2/I-FABP)	Mature enterocytes of the small intestine (highly expressed in the duodenum/jejunum), with low-level expression in colonic, renal and adipocytes (75)	Mediates the uptake, intracellular trafficking and metabolism of long-chain fatty acids; serves as a sensitive biomarker for intestinal epithelial injury; may regulate the proliferation and differentiation of intestinal stem cells (76)
Heart-type (FABP3)	Cardiomyocytes, skeletal muscle cells (77)	Supports fatty acid-dependent energy metabolism and supplies energy for cardiac and skeletal muscles (74)
Adipocyte-type (FABP4)	Adipocytes, macrophages (14)	Regulates lipid storage; participates in macrophage-mediated inflammatory responses (14)
Epidermal-type (FABP5)	Epidermal cells, various epithelial cells (74)	Mediates the trafficking of lipophilic molecules and participates in epidermal lipid metabolism and cell proliferation (14)

Despite their tissue specificity, FABP family members share a conserved three-dimensional structure. Most isoforms possess a characteristic β-barrel configuration that forms a central hydrophobic cavity for ligand binding (18). This structural framework underlies their shared ability to solubilize and transport fatty acids and other lipophilic molecules within the aqueous cytoplasmic environment. However, the disease relevance of individual FABPs depends on cell type, ligand availability, interacting signaling pathways, and the experimental or clinical context in which they are studied.

#### 3.1.2 Molecular structure and genetic characteristics of FABP2

FABP2, also known as intestinal fatty acid-binding protein (I-FABP), is the intestine-specific isoform of the FABP family. In humans, the *FABP2* gene is located on chromosome 4q28–q31 and encodes a 132–amino acid protein with a molecular weight of approximately 15 kDa (19).

Crystallographic analyses demonstrate that FABP2 adopts a compact globular structure composed of 10 antiparallel β-strands arranged into a β-barrel, forming a central hydrophobic cavity for fatty acid binding (20). This conserved FABP fold provides the structural basis for reversible binding and intracellular trafficking of long-chain fatty acids. Ligand entry and release are thought to involve a dynamic portal region formed by two short α-helices and adjacent loop segments, although the precise conformational changes may vary according to ligand type and experimental conditions (21).

One of the most extensively studied genetic variants of FABP2 is the Ala54Thr polymorphism (rs1799883), which results in an alanine-to-threonine substitution at codon 54 (22). Functional studies have suggested that the Thr54 variant may alter FABP2 fatty acid-binding properties and intestinal lipid handling, while human association studies have linked this polymorphism to metabolic traits such as insulin resistance, obesity, type 2 diabetes, and cardiovascular risk (22, 23). However, these associations are not entirely consistent across populations, and the variant should be interpreted as a potential genetic modifier rather than a deterministic cause of metabolic disease. Thus, evidence regarding Ala54Thr includes both experimental observations on ligand-binding behavior and clinical genetic association studies.

#### 3.1.3 Tissue distribution and cellular localization of FABP2

FABP2 exhibits a highly restricted tissue distribution and is predominantly expressed in mature enterocytes of the small intestine, particularly within villus epithelial cells (24). Within the small intestine, FABP2 expression is highest in the duodenum and jejunum and comparatively lower in the ileum, paralleling regional differences in fatty acid absorption efficiency. Although primarily considered a small intestinal protein (25), low-level expression has also been reported in other tissues, including the colon, kidney, and adipocytes; however, the biological significance of these low-level signals remains less well-defined (26).

At the cellular level, FABP2 is enriched in the apical cytoplasm of enterocytes, adjacent to the brush border membrane, where dietary fatty acid uptake occurs (21). This localization positions FABP2 to bind fatty acids shortly after their entry into enterocytes, buffer potentially cytotoxic concentrations of unbound fatty acids, and facilitate their intracellular trafficking toward metabolic destinations such as oxidation, esterification, or lipid-mediated signaling pathways (27, 28). Thus, the regional and subcellular distribution of FABP2 is consistent with a role in intestinal lipid handling.

#### 3.1.4 Physiological functions of FABP2

The principal physiological function of FABP2 is to participate in the intracellular handling of long-chain fatty acids within small intestinal epithelial cells (29, 30). After dietary triglycerides are hydrolyzed by pancreatic lipases into free fatty acids and monoacylglycerols, these lipids are absorbed by enterocytes through passive diffusion across the membrane and protein-facilitated transport involving molecules such as CD36 and Fatty acid transport protein 4 (FATP4) (31). After entering the cytoplasm, long-chain fatty acids can bind to FABP2 with high affinity (32, 33). This binding is thought to help buffer the intracellular pool of unbounded fatty acids, support continued fatty acid influx by maintaining a favorable concentration gradient, and reduce potential lipotoxic effects caused by excessive free fatty acids. As a cytosolic lipid chaperone, FABP2 is generally considered to facilitates the intracellular trafficking of fatty acids toward metabolic destinations, including the endoplasmic reticulum for triglyceride re-esterification and, under specific metabolic conditions, mitochondria for β-oxidation (33, 34).

Beyond intracellular fatty acid trafficking, FABP2 may influence lipid-dependent gene regulation by controlling the availability of fatty acid ligands for nuclear receptors. The strongest evidence relates to peroxisome proliferator-activated receptor α (PPARα), a lipid-sensing nuclear receptor that regulates genes involved in fatty acid oxidation and lipid metabolism. FABP2 has been reported to bind PPARα ligands, but its ligand-binding kinetics and capacity to promote nuclear receptor activation differ from those of FABP1 (6, 35). Hughes et al. (36) showed that both FABP1 and FABP2 could bind PPARα ligands; however, FABP1 more efficiently promoted PPARα transcriptional activation, whereas the effect of FABP2 was comparatively weaker. These findings suggest that FABP2 may participate in PPARα-related lipid metabolic signaling by modulating ligand availability, but they do not establish that FABP2 broadly activates PPAR pathways or directly regulates inflammatory or epithelial repair programs. Therefore, proposed links between FABP2 and PPARγ- or PPARβ/δ-mediated inflammatory or epithelial repair pathways should be interpreted as more indirect. Evidence from other FABP isoforms suggests that FABP-mediated lipid delivery to the nucleus can influence PPARγ activity (7, 8), and PPARβ/δ signaling has been implicated in epithelial repair and mucosal homeostasis (11–13). However, whether FABP2 directly delivers ligands to PPARγ or modulates PPARβ/δ-driven epithelial restitution in enterocytes remains insufficiently demonstrated. Thus, the proposed links between FABP2, intestinal barrier integrity, epithelial renewal, and inflammatory responses are biologically plausible but remain largely inferential and require validation in disease-relevant epithelial models, animal models of intestinal inflammation, and human IBD tissues.

Taken together, FABP2 has well-supported roles in intracellular fatty acid binding and trafficking in enterocytes, whereas its broader functions in nuclear receptor signaling, epithelial repair, and intestinal inflammation are supported mainly by experimental or indirect evidence. To improve readability, the molecular characteristics and functional relevance of FABP2 are summarized separately. Structural, genetic, and localization features are presented in Table 2A, whereas physiological functions and clinical relevance are summarized in Table 2B.

**Table 2A:** Structural and genetic characteristics of FABP2.

Category	Key feature	Molecular details	Evidence type and interpretation
Gene localization	Chromosomal location	The human *FABP2* gene is mapped to chromosome 4q28–q31 (36).	Genomic annotation; descriptive molecular evidence.
Protein features	Size and structure	FABP2 is composed of 132 amino acids and has a molecular weight of approximately 15 kDa. It adopts a conserved FABP fold characterized by a β-barrel hydrophobic cavity formed mainly by 10 antiparallel β-strands, with a dynamic portal region involved in ligand entry and release (20).	Structural and biochemical evidence; supports ligand-binding capacity.
Genetic characteristics	Ala54Thr polymorphism	The Ala54Thr variant (rs1799883) may alter fatty acid-binding properties and intestinal lipid handling and has been associated with obesity, type 2 diabetes, insulin resistance, and cardiovascular risk in some human studies (22).	Functional and human association evidence; associations vary among populations and should not be interpreted as deterministic.
Cellular localization	Subcellular distribution	FABP2 is enriched in the cytoplasm of absorptive enterocytes, particularly near the apical region adjacent to the brush border membrane, where dietary fatty acid uptake occurs (21).	Expression and cell-biological evidence; supports a role in post-uptake intracellular fatty acid handling.

**Table 2B:** Physiological functions and clinical relevance of FABP2.

Category	Core function or relevance	Key details	Evidence type and interpretation
Lipid metabolism	Intracellular fatty acid handling	FABP2 binds long-chain fatty acids, commonly including 16- to 20-carbon fatty acids, and may facilitate their intracellular trafficking toward metabolic destinations such as the endoplasmic reticulum for triglyceride re-esterification and, under certain metabolic conditions, mitochondria for β-oxidation (36).	Biochemical and physiological evidence; mitochondrial delivery and metabolic channeling may be context-dependent.
Signaling and gene regulation	Lipid-mediated nuclear receptor signaling	FABP2 may participate in lipid-mediated signaling by modulating ligand availability for nuclear receptors, particularly PPARα. Its direct involvement in PPARγ- or PPARβ/δ-mediated inflammatory or epithelial repair pathways remains insufficiently established (6).	Experimental and inferential evidence; direct pathway-specific validation remains limited.
Biomarker characteristics	Marker of epithelial injury	FABP2 can be released into the circulation and/or intestinal lumen after enterocyte injury and has been investigated as a biomarker of intestinal mucosal damage (37).	Human clinical and experimental biomarker evidence; potentially sensitive for enterocyte injury but not specific to IBD or a single disease process.

### 3.2 The role of FABP2 in multisystem diseases

#### 3.2.1 Digestive system diseases

In the digestive system, FABP2 has been studied primarily as an enterocyte-enriched protein involved in lipid handling and as a biomarker of epithelial injury. FABP2 is mainly expressed in mature intestinal epithelial cells, especially those at the tip of the intestinal villi, a region highly susceptible to inflammatory injury (37, 38). Upon epithelial damage or loss of cell membrane integrity, FABP2 can be rapidly released into the circulation and intestinal lumen, making it a sensitive indicator of intestinal epithelial cell injury and impaired barrier function (39). Human clinical studies have reported increased circulating FABP2 levels in several conditions associated with intestinal mucosal damage, including intestinal ischemia, necrotizing enterocolitis, hemorrhagic shock, abdominal trauma, celiac disease, and IBD-related mucosal injury (40–43). In acute pancreatitis, elevated FABP2 levels have also been associated with intestinal barrier dysfunction, indicating disease severity and potential complications (24). These findings support the clinical utility of FABP2 as a marker reflecting the presence or extent of intestinal epithelial injury. However, because FABP2 release occurs in diverse forms of enterocyte damage, it should be regarded as an injury-associated biomarker rather than a disease-specific marker for IBD or any single gastrointestinal disorder.

In colorectal cancer, altered FABP2 expression patterns has been reported in tissues and has been associated with tumor development or progression in some studies (44, 45). Experimental and bioinformatic analyses suggest that FABP2 may be linked to tumor cell lipid metabolism, proliferation, and invasion through lipid-related signaling pathways (45). Nevertheless, the functional contribution of FABP2 to colorectal tumorigenesis remains incompletely defined, and its potential as a therapeutic target requires further validation in mechanistic models and clinical cohorts.

#### 3.2.2 Metabolic diseases

FABP2 has been investigated in metabolic diseases, particularly obesity, insulin resistance, and type 2 diabetes, because of its role in intestinal long-chain fatty acid handling. The strongest human evidence is derived mainly from genetic association studies of the FABP2 Ala54Thr polymorphism and clinical studies evaluating circulating FABP2 as a marker of intestinal epithelial injury or metabolic disturbance. Several studies have associated the Thr54 variant with altered lipid absorption, postprandial lipemia, insulin resistance, obesity, type 2 diabetes, or cardiovascular risk; however, these associations are not fully consistent across populations and may be influenced by ethnicity, diet, adiposity, and other metabolic risk factors (22).

As described above, FABP2 participates in enterocyte fatty acid handling. Therefore, genetic or functional alterations in FABP2 may influence postprandial lipid metabolism and systemic metabolic traits, although direct causal evidence in humans remains limited. Mechanistically, excess lipid delivery has been proposed to promote the accumulation of lipotoxic intermediates, including diacylglycerols and ceramides, which may impair insulin signaling pathways, while potentially activating pro-inflammatory cascades such as NF-κB, resulting in chronic low-grade inflammation (9). In addition, lipid overload associated with FABP2 dysfunction has been linked to disruption of intestinal barrier integrity and increased permeability, facilitating lipopolysaccharide translocation and metabolic endotoxemia, which further exacerbates systemic inflammation and metabolic dysfunction (46). Beyond its metabolic role, FABP2 may influence nuclear receptor signaling by modulating lipid availability for PPAR pathways, thereby affecting transcriptional programs involved in lipid metabolism, glucose homeostasis, and inflammation (7). However, these mechanisms are supported largely by experimental evidence from lipid metabolism studies and by indirect inference, rather than by direct proof that FABP2 drives metabolic disease progression in humans.

Overall, current evidence supports a role for FABP2 in intestinal fatty acid handling and suggests potential links with metabolic traits. However, FABP2 should be described as a candidate biomarker or genetic modifier of metabolic dysfunction rather than an established causal driver or validated therapeutic target. Further studies combining human cohorts, controlled dietary interventions, FABP2 gain- or loss-of-function models, and tissue-specific mechanistic analyses are needed to clarify its causal contribution to metabolic diseases.

#### 3.2.3 Cardiovascular diseases

The relationship between FABP2 and cardiovascular diseases has been investigated mainly from two perspectives: as a marker of intestinal injury during cardiovascular stress and as a potential genetic or metabolic modifier of cardiovascular risk. In acute myocardial infarction and other severe cardiovascular conditions, elevated circulating FABP2 levels may reflect intestinal hypoperfusion, ischemia-reperfusion injury, or impaired epithelial barrier integrity rather than direct myocardial injury (47, 48). Therefore, in this clinical context, FABP2 is best interpreted as a marker of enterocyte injury and possible multi-organ dysfunction, which may be associated with disease severity and poor prognosis.

FABP2 may also be indirectly linked to cardiovascular risk through its role in intestinal lipid handling. By binding long-chain fatty acids and participating in intracellular lipid trafficking in enterocytes, FABP2 could influence postprandial lipid metabolism, circulating triglyceride-rich lipoproteins, and downstream atherogenic risk factors (9, 21). However, direct evidence that FABP2 dysfunction independently causes dyslipidemia or atherosclerosis in humans remains limited.

Inflammatory mechanisms have also been proposed. Altered intestinal lipid handling, epithelial injury, or increased gut permeability may contribute to systemic low-grade inflammation, which is relevant to vascular inflammation and plaque instability. Some studies have associated FABP2-related changes or the Ala54Thr polymorphism with inflammatory mediators such as Tumor Necrosis Factor Alpha (TNF-α), IL-6, and IL-1β, as well as with cardiovascular outcomes including coronary heart disease and stroke (23, 49). Nevertheless, these findings are largely associative and may be influenced by metabolic comorbidities, diet, obesity, and population-specific genetic background. Thus, FABP2 should currently be considered a potential biomarker or modifier of cardiovascular risk rather than a proven causal mediator or validated therapeutic target.

#### 3.2.4 Neurological, pulmonary, and renal diseases

In neurological diseases, the potential relevance of FABP2 is largely indirect. In Parkinson's disease, the “dual-hit” hypothesis, which proposes that α-synuclein pathology may originate in the gut and subsequently spread to the brain (50), has gained increasing attention. In this context, intestinal barrier dysfunction, gut inflammation, and altered lipid metabolism have been proposed to contribute to gut-brain axis disturbances. However, direct evidence linking FABP2 dysfunction to α-synuclein pathology, neuroinflammation, or Parkinson's disease progression remains limited. Similarly, in Alzheimer's disease, intestinal inflammation, microbiota alterations, and lipid metabolic dysregulation have been be associated with cognitive decline (51, 52). Nevertheless, the specific contribution of FABP2 to these processes remains speculative and should be considered a mechanistic hypothesis rather than an established disease mechanism. In pulmonary diseases, particularly acute lung injury and acute respiratory distress syndrome (ARDS), elevated plasma FABP2 levels have been reported in association with intestinal dysfunction, systemic inflammation, and disease severity (53–55). In renal diseases, especially acute kidney injury (AKI), FABP2 has been investigated as a marker of intestinal ischemia-reperfusion injury or gut barrier impairment, which may occur during shock, sepsis, major surgery or other states of systemic hypoperfusion (46, 56). Thus, the association between FABP2 and AKI may reflect gut-kidney crosstalk and multi-organ dysfunction rather than a direct renal function of FABP2. Further studies are needed to determine whether FABP2 is merely a biomarker of intestinal injury in these conditions or whether it actively contributes to systemic inflammatory and organ specific injury pathways.

### 3.3 The role of FABP2 in inflammatory bowel disease

#### 3.3.1 FABP2 as a biomarker in IBD

In IBD specifically, the best supported clinical role of FABP2 is an injury associated biomarker of enterocyte damage rather than an IBD-specific diagnostic marker (57). Human studies have reported increased FABP2 levels in patients with active IBD, with some evidence linking FABP2 concentrations to clinical disease activity indices, including the Crohn's Disease Activity Index (CDAI) and the Mayo Clinical Score for ulcerative colitis (58, 59). These associations are biologically consistent with the enterocyte-enriched expression and rapid release of FABP2 after epithelial injury; however, the strength and consistency of these correlations vary across studies. Because FABP2 release may occur early after enterocyte injury, it has been proposed as a candidate marker for early epithelial damage (60). Nevertheless, whether FABP2 can reliably precede clinical symptoms, predict flare, or independently guide treatment decisions remains unclear and requires prospective validation.

Similarly, declining FABP2 levels during treatment could theoretically reflect reduced epithelial injury or mucosal recovery. However, direct evidence linking FABP2 dynamics to endoscopic mucosal healing remains limited, and standardized cut-off values, assay methods, and sampling strategies have not yet been established. Therefore, FABP2 is best considered a complementary biomarker rather than a replacement for established tools. In this context, fecal calprotectin remains the better-validated non-invasive biomarker IBD disease activity, relapse risk, as well as endoscopic inflammation (61, 62). FABP2 may provide distinct biological information by reflecting enterocyte damage and barrier dysfunction rather than neutrophil-driven inflammation (21). Combined assessment of fecal calprotectin and FABP2 may therefore offer complementary insight into inflammatory burden and epithelial injury, but this approach requires further validation before routine clinical application.

#### 3.3.2 Molecular mechanisms in IBD pathogenesis

##### 3.3.2.1 Intestinal barrier dysfunction

Intestinal epithelial barrier dysfunction is a central feature of IBD pathogenesis and involves increased intestinal permeability, altered tight junction integrity, epithelial apoptosis or shedding, and immune cell mediated epithelial injury. This process can amplify mucosal immune activation and sustain intestinal inflammation. Barrier dysfunction in IBD involves several interrelated processes: (1) Tight junction proteins: inflammatory cytokines and epithelial stress can alter the expression, localization, or function of tight junction proteins, including occludin, claudin-1, and zonula occludens-1 (ZO-1), thereby increasing paracellular permeability; (2) Intestinal epithelial cell apoptosis: excessive apoptosis or shedding of intestinal epithelial cells can compromise epithelial continuity; (3) Inflammatory cell infiltration: infiltration of immune cells, including neutrophils, macrophages, and T cells, can further aggravate epithelial injury through cytokine production, oxidative stress, and protease release. However, current evidence does not establish that FABP2 itself downregulates tight junction proteins, induces epithelial apoptosis, or initiates inflammatory-cell infiltration in IBD.

##### 3.3.2.2 Lipid metabolism disorders

Altered lipid metabolism is increasingly recognized as a feature of IBD pathogenesis and mucosal inflammation. Human metabolomic studies and experimental colitis models have reported changes in fatty acid profiles, eicosanoid metabolism, mitochondrial function, and epithelial energy homeostasis in inflamed intestinal tissues (34, 63, 64).

Given its established role in enterocyte lipid handling, FABP2 may be relevant to lipid metabolic remodeling in inflamed intestinal mucosa (65). However, current evidence more strongly supports an association between FABP2 expression or release and epithelial injury than a direct causal role for FABP2 in systemic lipid dysregulation in IBD. Several mechanisms have been proposed to link disturbed intestinal lipid metabolism with chronic mucosal inflammation. First, epithelial damage and altered FABP2 expression may impair intracellular handling of dietary long-chain fatty acids, although whether this translates into clinically meaningful defects in fatty acid absorption in IBD remains to be established. Second, dysregulated lipid metabolism may alter the generation of bioactive lipid mediators, including prostaglandins and leukotrienes, which can amplify or modulate inflammatory responses. Third, reduced fatty acid utilization and mitochondrial dysfunction may compromise epithelial energy homeostasis, thereby weakening barrier maintenance and tissue repair. These mechanisms are biologically plausible and supported by broader lipid metabolism studies, but direct FABP2-specific evidence in human IBD remains limited.

##### 3.3.2.3 FABP2–microbiota interactions

Recent studies have suggested a potential link between FABP2, intestinal epithelial injury, and gut microbiota-related inflammatory processes, particularly in Crohn's disease (66). However, current evidence in this area remains limited. Because FABP2 is primarily a cytosolic enterocyte protein, its presence in the intestinal lumen is generally interpreted as a consequence of epithelial damage rather than active secretion. Therefore, FABP2 is more plausibly involved in host–microbe crosstalk after epithelial injury has occurred, rather than as an upstream regulator of microbiota composition.

The best-characterized FABP2-specific microbiota mechanism involves *Enterococcus faecalis*. Experimental studies have reported that FABP2 released from damaged intestinal epithelial cells can be recognized by *E. faecalis* through the EF3041 protein, potentially activating quorum-sensing responses and promoting virulence-related behaviors such as biofilm formation (67). These findings provide a mechanistic example of how epithelial injury-derived FABP2 could modify host–microbe interactions. However, this FABP2–*E. faecalis* axis is currently based on limited experimental evidence. Independent replication remains insufficient, and direct human IBD evidence linking luminal FABP2 levels to *E. faecalis* abundance, EF3041 expression, biofilm formation, or disease activity is still lacking. Comparable FABP2-specific interactions with other bacterial taxa have also not been clearly demonstrated.

Therefore, FABP2–microbiota interactions should be viewed as an emerging, taxon-specific mechanistic hypothesis rather than a broadly established pathway in IBD pathogenesis. Broader links among FABP2, dysbiosis, microbial metabolites, and mucosal inflammation remain indirect and hypothesis-generating. Future studies integrating mucosal or luminal FABP2 measurement with strain-level microbiome profiling, EF3041 detection, microbial virulence assays, and clinical disease activity data are needed to determine whether this pathway is reproducible and clinically relevant.

##### 3.3.2.4 Regulation of intestinal stem cells

Intestinal stem cells are essential for epithelial renewal and mucosal repair after inflammatory injury. In IBD, chronic inflammation, cytokine exposure, oxidative stress, and metabolic remodeling can impair intestinal stem cell function, thereby delaying epithelial regeneration and contributing to persistent mucosal damage (68). Broader studies of intestinal epithelial renewal indicate that stem-cell activity is metabolically regulated, including through fatty-acid oxidation and lipid-sensitive signaling pathways (69). However, these findings do not demonstrate that FABP2 directly regulates intestinal stem-cell biology.

**Figure 2:**
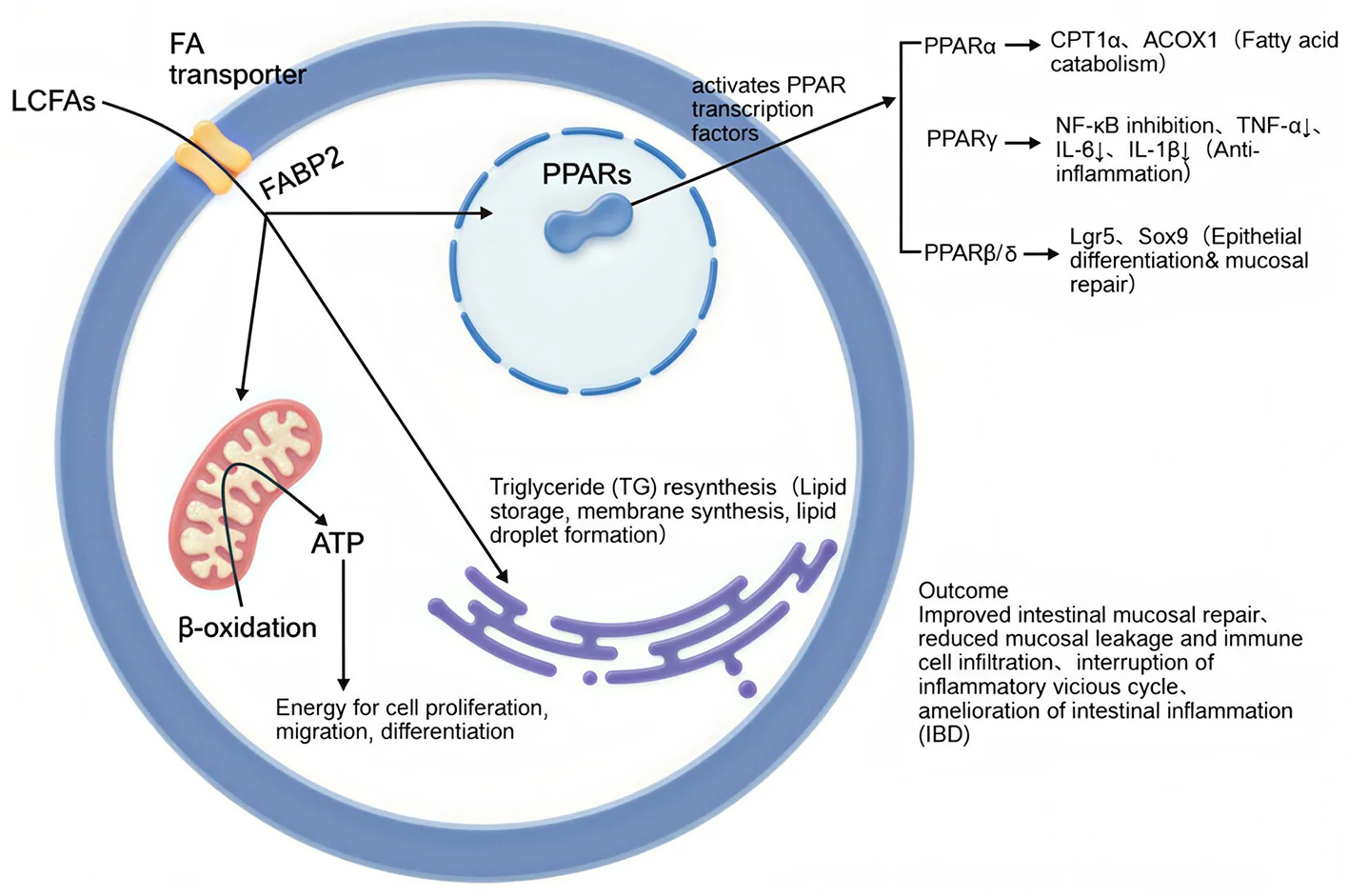
Proposed mechanisms by which FABP2 may influence intestinal mucosal repair and inflammatory responses in inflammatory bowel disease.

Current evidence does not establish a direct FABP2-specific role in intestinal stem-cell proliferation, differentiation, Lgr5/Sox9 expression, organoid-forming capacity, or mucosal regeneration. Thus, any proposed effect of FABP2 on Lgr5-, Sox9- (70), or other stem cell-associated pathways should be interpreted as a mechanistic hypothesis rather than a demonstrated regulatory relationship. FABP2 is mainly expressed in mature absorptive enterocytes rather than being established as an intestinal stem-cell marker. As schematically summarized in Figure 2, proposed links between FABP2, lipid metabolism, intestinal stem-cell activity, and epithelial repair should be interpreted as indirect hypotheses based on broader lipid-metabolic biology, not as demonstrated FABP2-specific regulatory relationships. Direct testing would require FABP2 gain- and loss-of-function models, intestinal organoids, lineage-tracing systems, and experimental colitis models assessing stem-cell markers, lineage differentiation, and mucosal repair outcomes.

Proposed model illustrating the potential role of FABP2 in intestinal stem cell function and mucosal repair. FABP2 may influence intestinal stem cell proliferation and differentiation through its involvement in long-chain fatty acid transport, mitochondrial β-oxidation, and PPAR-related signaling pathways. These mechanisms may contribute to intestinal mucosal repair and inflammatory regulation, although further studies are required to validate these pathways.

##### 3.3.2.5 Inflammatory signaling pathways

In addition to intestinal barrier dysfunction, lipid metabolic remodeling, gut microbiota-related mechanisms, and the proposed effects on epithelial repair, FABP2 has also been hypothesized to participates in inflammatory signaling pathways that may be relevant to IBD pathogenesis (55, 71). However, most available evidence supports an association between FABP2, epithelial injury, and lipid metabolic disturbance rather than a direct regulatory role for FABP2 in canonical inflammatory pathways. Therefore, the following pathways should be interpreted as mechanistic hypotheses that require further validation in FABP2-specific experimental models.

- NF-κB signaling pathway: NF-κB is a central regulator of mucosal inflammation and controls the expression of multiple pro-inflammatory mediators, including TNF-α, IL-1β, and IL-6. In IBD, sustained NF-κB activation contributes to immune-cell recruitment, epithelial injury, and amplification of inflammatory responses. FABP2 may be indirectly linked to this pathway through its role in enterocyte lipid handling and epithelial integrity. Altered FABP2 expression or release may reflect epithelial damage, lipid stress, oxidative stress, or impaired barrier function, all of which can contribute to NF-κB activation. Conversely, maintenance of lipid homeostasis and barrier integrity could theoretically limit excessive NF-κB activation. Nevertheless, direct evidence that FABP2 itself activates or suppresses NF-κB signaling in IBD remains limited.
- PPAR signaling pathway: PPARs are lipid-sensitive nuclear receptors involved in lipid metabolism, epithelial homeostasis, and inflammatory regulation. Because FABP2 binds long-chain fatty acids, it may influence the intracellular availability of lipid ligands for PPAR signaling. Experimental studies provide stronger support for interactions between FABP2 and PPARα-related lipid metabolic signaling, whereas direct evidence for FABP2-mediated regulation of PPARγ or PPARβ/δ pathways in intestinal inflammation remains insufficient. Thus, the proposed FABP2-PPAR axis in IBD is biologically plausible but should be described as an indirect lipid-mediated mechanism rather than an established inflammatory pathway.
- Mitogen-Activated Protein Kinase (MAPK) signaling pathway: MAPK pathways, including Extracellular Signal-Regulated Kinase (ERK), c-Jun N-terminal kinase (JNK), and p38, regulate epithelial stress responses, cell survival, proliferation, and inflammatory mediator production. These pathways are activated in response to cytokines, oxidative stress, microbial products, and tissue injury in IBD. FABP2 has been proposed to influence MAPK signaling through changes in lipid metabolism, lipotoxic stress, or epithelial damage; however, direct FABP2-specific evidence is currently limited. In particular, whether FABP2 directly modulates MAPK-dependent epithelial proliferation, survival, or repair has not been established.

Overall, FABP2 may be connected to inflammatory signaling mainly through its enterocyte-specific role in fatty acid handling and as a marker of epithelial injury. Current evidence does not yet support FABP2 as a confirmed upstream regulator of NF-κB, PPARγ/PPARβ/δ, or MAPK signaling in IBD. Future studies using FABP2 knockdown or overexpression in intestinal epithelial cells, intestinal organoids, and experimental colitis models are needed to determine whether FABP2 actively regulates these pathways or primarily reflects epithelial injury and metabolic stress.

#### 3.3.3 Clinical applications in IBD

##### 3.3.3.1 Disease diagnosis and differential diagnosis

FABP2 has potential value as a complementary biomarker for identifying intestinal epithelial injury in patients with suspected IBD. Because FABP2 is released after enterocyte damage, elevated circulating or fecal FABP2 levels may support the presence of mucosal injury or barrier disruption. In contrast, irritable bowel syndrome (IBS) is generally considered a functional gastrointestinal disorder and is not typically characterized by overt mucosal injury, so FABP2 levels may be lower in IBS than in active IBD.

However, FABP2 should not be used as a stand-alone diagnostic or differential diagnostic marker for IBD. Increased FABP2 levels can occur in multiple gastrointestinal and systemic conditions involving enterocyte injury, including infectious enteritis, intestinal ischemia, celiac disease, trauma, surgery, shock, and critical illness. Therefore, FABP2 may help distinguish inflammatory or injury-associated intestinal conditions from functional disorders only when interpreted together with clinical symptoms, endoscopy, histology, imaging, fecal calprotectin, C-reactive protein, and other established diagnostic tools. Further prospective studies are needed to determine its diagnostic accuracy, optimal cut-off values, and added value beyond existing biomarkers.

##### 3.3.3.2 Disease monitoring and prognosis

Serial measurement of FABP2 levels may provide a non-invasive approach for monitoring enterocyte injury and barrier disruption in patients with IBD. Because FABP2 can be released rapidly after epithelial cell damage, changes in circulating or fecal FABP2 levels may reflect dynamic mucosal injury during active inflammation. Human clinical studies have suggested associations between FABP2 levels and disease activity, but its performance for longitudinal disease monitoring remains less well-established than that of fecal calprotectin, C-reactive protein, endoscopic assessment, and histological evaluation.

Potential clinical applications of FABP2 include early detection of epithelial injury during disease flare, assessment of treatment response, and evaluation of relapse risk. However, these applications remain investigational. It has not yet been clearly established whether FABP2 rises before clinical symptoms, predicts relapse independently of established biomarkers, or reliably reflects endoscopic mucosal healing. In addition, standardized assays, clinically meaningful cut-off values, sampling intervals, and interpretation frameworks are still lacking.

Therefore, FABP2 may be considered a candidate complementary biomarker for IBD monitoring and prognosis, particularly for assessing epithelial injury rather than inflammatory burden alone. Prospective longitudinal studies are needed to determine whether serial FABP2 measurement adds clinically useful information beyond fecal calprotectin, C-reactive protein, clinical activity indices, and endoscopic findings.

##### 3.3.3.3 Therapeutic target development

The potential involvement of FABP2 in epithelial lipid handling, barrier injury, and inflammatory regulation has raised interest in its possible relevance to therapeutic development for IBD. However, FABP2 should currently be regarded as an exploratory target rather than a validated therapeutic target. Existing evidence mainly supports its role as an enterocyte lipid-binding protein and a biomarker of epithelial injury, whereas direct evidence that pharmacological or genetic modulation of FABP2 improves mucosal healing, reduces inflammation, or restores intestinal barrier function in IBD remains limited.

Several FABP2-related therapeutic concepts have been proposed based on its biological functions and disease associations. These include small-molecule modulators of FABP2 ligand binding or activity, intestinal-targeted delivery systems, microbiota-directed strategies, and dietary interventions aimed at modifying fatty acid composition or lipid metabolic stress (72, 73). However, these strategies remain largely conceptual. At present, it is unclear whether directly increasing, inhibiting, or otherwise modifying FABP2 activity would be beneficial in IBD, because FABP2 may reflect epithelial injury rather than drive the disease process.

Future therapeutic studies should first clarify whether FABP2 has a causal role in IBD pathogenesis. This will require FABP2 gain- and loss-of-function experiments in intestinal epithelial cells, organoids, and experimental colitis models, followed by validation in human IBD tissues and prospective clinical cohorts. Only after such mechanistic and translational evidence is established can FABP2 be considered a credible therapeutic target for mucosal repair or inflammatory modulation in IBD.

##### 3.3.3.4 Summary of current evidence regarding FABP2 in IBD

Given the heterogeneity of current evidence, the available studies regarding FABP2 in IBD are summarized according to evidence category, study type, population or experimental model, major findings, and potential clinical implications. This structured summary distinguishes human clinical evidence from animal, experimental, and hypothesis-generating mechanistic studies, thereby helping clarify the strength and translational relevance of each proposed role of FABP2 in IBD. As shown in Table 3, the strongest evidence currently supports FABP2 as a marker of enterocyte injury, whereas its roles in lipid metabolic remodeling, microbiota interactions, inflammatory signaling, intestinal stem cell regulation, and therapeutic targeting remain less well-established and require further validation.

**Table 3:** Summary of available evidence regarding FABP2 in IBD.

Evidence category	Study type	Population/model	Key findings	Clinical implications	References
Epithelial injury biomarker	Clinical studies	CD/UC patients; IBD cohorts	FABP2 increases with enterocyte injury and may reflect active mucosal damage.	Candidate complementary marker; not IBD-specific.	(24, 57, 58, 60)
Gut barrier dysfunction	Clinical and experimental studies; reviews	IBD patients; barrier injury models	FABP2 release indicates epithelial damage and impaired barrier integrity.	May help assess gut barrier injury with other markers.	(19, 27, 55, 58, 60, 71)
Comparison with established biomarkers	Clinical biomarker studies	IBD cohorts; intestinal injury cohorts	Fecal calprotectin reflects inflammatory activity; FABP2 reflects epithelial injury.	Potentially complementary to fecal calprotectin, not a replacement.	(37, 58, 61, 62)
Lipid metabolism	Biochemical, animal, and mechanistic studies	Enterocytes; IFABP-null mice; IBD tissues	FABP2 mediates fatty acid handling; IBD shows altered intestinal lipid metabolism.	Supports possible metabolic link to mucosal inflammation.	(6, 6, 21, 31–36, 63–65)
Microbiota interaction	Experimental and translational studies	*E. faecalis* models; CD-related settings	Luminal FABP2 may activate *E. faecalis* quorum sensing and virulence.	Emerging mechanism; needs human validation.	(1, 3–5, 67)
Inflammatory signaling	Experimental and inferential evidence	Epithelial and colitis models	FABP2 may link lipid stress with PPAR, NF-κB, and MAPK signaling.	Hypothesis-generating; causal evidence limited.	(7–13, 35, 36, 66, 71)
Stem cell regulation and repair	Related experimental evidence; hypothesis	ISC and epithelial renewal models	FAO and lipid signaling affect renewal; direct FABP2-ISC evidence is limited.	Requires FABP2-specific organoid and *in vivo* validation.	(68–70)
Translational applications	Reviews and emerging intervention studies	Biomarker, nutrition, and microbiota contexts	FABP2 is a candidate biomarker and exploratory therapeutic target.	Use in multimodal assessment; target role unvalidated.	(24, 35, 59, 72, 73)

CD, Crohn's disease; UC, ulcerative colitis; IBD, inflammatory bowel disease; ISC, intestinal stem cell; FAO, fatty acid oxidation. References indicate representative studies.

## 4 Strengths, limitations, and future perspectives

### 4.1 Strengths and limitations of current evidence

Current evidence regarding FABP2 in IBD has several strengths. FABP2 has a well-defined biological basis as an intestine-enriched cytosolic lipid-binding protein that is rapidly released after enterocyte injury. This provides a plausible rationale for its use as a biomarker of intestinal epithelial damage. In addition, studies from clinical cohorts, experimental models, biochemical analyses, and microbiota-related investigations collectively suggest that FABP2 may connect epithelial lipid handling, barrier dysfunction, host-microbe interactions, and inflammatory responses. The availability of both human observational data and experimental evidence provides a foundation for further translational research.

However, important limitations should be acknowledged. Human studies remain relatively limited, especially prospective longitudinal studies with paired endoscopic, histological, and biomarker outcomes. Much of the mechanistic evidence is derived from experimental or preclinical models, and several proposed pathways, including FABP2 involvement in intestinal stem cell regulation, PPARγ/PPARβ/δ signaling, microbiota-derived metabolites, and mucosal repair, remain largely inferential. Direct causal evidence demonstrating that FABP2 modulation alters IBD onset, progression, inflammation, or mucosal healing is still insufficient.

Methodological heterogeneity also limits interpretation across studies. Differences in sample type, assay platform, disease phenotype, disease activity definitions, treatment exposure, population characteristics, and analytical cut-off values make it difficult to compare findings or establish standardized clinical thresholds. In addition, FABP2 is not specific to IBD and may be elevated in other conditions associated with enterocyte injury, including intestinal ischemia, infection, celiac disease, trauma, surgery, shock, and critical illness. Therefore, FABP2 should currently be interpreted as a candidate complementary biomarker and hypothesis-generating mechanistic molecule rather than a validated stand-alone diagnostic marker or therapeutic target.

### 4.2 Technical and clinical challenges

Despite the potential value of FABP2 as a candidate biomarker of intestinal epithelial injury in IBD, several technical and clinical challenges must be addressed before routine clinical application. First, FABP2 measurement lacks assay standardization across laboratories. Differences in sample type, including serum, plasma, urine, or stool, as well as detection platforms, calibration standards, antibody specificity, storage conditions, and reporting units may contribute to variability among studies. This heterogeneity makes it difficult to compare results directly and limits the establishment of clinically meaningful thresholds. Second, reference intervals and disease-relevant cut-off values remain insufficiently defined. Large-scale, multicenter studies are needed to determine whether FABP2 thresholds vary according to age, sex, ethnicity, diet, body mass index, comorbidities, medication exposure, and disease phenotype. Because FABP2 reflects enterocyte injury rather than IBD-specific inflammation, its interpretation may also be confounded by infections, ischemia, surgery, trauma, critical illness, celiac disease, and other conditions associated with intestinal epithelial damage. Therefore, FABP2 is unlikely to function as a stand-alone diagnostic marker and should be evaluated as part of a multimodal biomarker panel. Third, the longitudinal performance of FABP2 requires further validation. Although clinical studies suggest associations between FABP2 levels and mucosal injury or disease activity, it remains unclear whether FABP2 can reliably detect early flare, predict relapse, monitor therapeutic response, or reflect endoscopic mucosal healing beyond established biomarkers such as fecal calprotectin and C-reactive protein. Prospective studies with standardized sampling intervals, paired endoscopic and histological outcomes, and predefined analytical cut-offs are required.

Translating FABP2 into a therapeutic target faces even greater uncertainty. Current evidence supports FABP2 mainly as an enterocyte lipid-binding protein and injury-associated biomarker, whereas direct evidence that modulating FABP2 improves intestinal inflammation or mucosal repair in IBD remains limited. FABP2-targeted interventions therefore remain largely theoretical and would require clarification of whether FABP2 is a causal mediator, protective adaptive response, or passive marker of epithelial injury. Additional challenges include achieving intestine-specific delivery, maintaining drug stability in the gastrointestinal tract, defining appropriate dosing strategies, and avoiding unintended disruption of dietary lipid absorption, epithelial metabolism, or systemic metabolic homeostasis.

### 4.3 Mechanistic gaps

Several key mechanistic questions regarding the role of FABP2 in IBD remain unresolved. First, it is still unclear whether FABP2 is an active mediator of IBD pathogenesis or primarily a marker of enterocyte injury. Most clinical studies show associations between FABP2 levels and intestinal epithelial damage, whereas direct causal evidence from FABP2-specific gain- or loss-of-function models remains limited. Clarifying this distinction is essential before FABP2 can be considered a therapeutic target.

Second, the proposed relationship between FABP2 and intestinal stem cell regulation remains largely hypothetical. Although lipid metabolism, fatty acid oxidation, and PPAR signaling are known to influence epithelial renewal, direct evidence that FABP2 regulates intestinal stem cell proliferation, differentiation, organoid-forming capacity, or markers such as Lgr5 and Sox9 is insufficient. Future studies should test this possibility using intestinal organoids, lineage-tracing approaches, FABP2 knockdown or overexpression systems, and experimental colitis models.

Third, FABP2-microbiota interactions require broader investigation. The reported interaction between FABP2 and *Enterococcus faecalis* provides a specific experimental example, but it remains unknown whether FABP2 influences broader microbial community structure, bacterial virulence programs, short-chain fatty acid production, or mucosal immune activation in human IBD. Integrated studies combining microbiome profiling, mucosal transcriptomics, luminal FABP2 measurement, and disease activity assessment are needed.

Fourth, the segment-specific and cell-type-specific functions of FABP2 remain incompletely defined. FABP2 is predominantly expressed in small intestinal enterocytes, whereas IBD affects different intestinal regions in UC and CD. Therefore, the relevance of FABP2 may differ between small intestinal Crohn's disease, colonic Crohn's disease, and ulcerative colitis. Spatial transcriptomics, single-cell analyses, and paired tissue studies from inflamed and non-inflamed intestinal regions could help clarify these context-dependent roles.

Finally, the potential involvement of FABP2 in extra-intestinal manifestations of IBD remains speculative. Elevated FABP2 levels in systemic diseases may reflect intestinal barrier injury and systemic inflammation rather than direct pathogenic effects in extra-intestinal tissues. Future human studies should distinguish whether FABP2 is a biomarker of gut-derived epithelial injury, a mediator of systemic inflammatory pathways, or an indirect correlate of disease severity.

### 4.4 Future directions

Despite these challenges, future research should further clarify the clinical and biological relevance of FABP2 in IBD through integrated clinical, genetic, mechanistic, and multi-omics approaches. Prospective clinical studies are needed to evaluate the diagnostic and prognostic value of FABP2 in comparison with established markers such as fecal calprotectin, C-reactive protein, endoscopic activity scores, and histological indices. Multiplex biomarker panels combining FABP2 with inflammatory markers, epithelial injury markers, and barrier function indicators may improve the assessment of disease activity, mucosal injury, treatment response, and relapse risk. In clinical practice, FABP2 may be particularly useful as a complementary marker reflecting enterocyte injury, rather than as a replacement for established inflammatory biomarkers.

FABP2 may also contribute to future patient stratification strategies. Integrated models combining FABP2 levels with clinical variables, disease location, endoscopic severity, histological activity, fecal calprotectin, C-Reactive Protein (CRP), microbiota profiles, lipidomic signatures, and genetic variants may help identify patients with predominant epithelial injury, barrier dysfunction, or metabolic-inflammatory phenotypes. Such stratification could support individualized monitoring strategies and may help predict relapse risk, incomplete mucosal healing, or suboptimal therapeutic response.

Genotype-phenotype studies may help determine whether FABP2 genetic variants, particularly Ala54Thr, influence intestinal lipid metabolism, epithelial injury, disease phenotype, or treatment outcomes in IBD. Such studies should include ethnically diverse populations, dietary information, metabolic profiles, and disease-location stratification to clarify whether FABP2 variants contribute meaningfully to inter-individual differences in disease course.

Mechanistic studies should directly address whether FABP2 is a causal mediator or primarily a biomarker of epithelial injury. FABP2 knockdown, knockout, or overexpression models in intestinal epithelial cells, intestinal organoids, and experimental colitis models would help define its effects on fatty acid handling, barrier integrity, inflammatory signaling, epithelial repair, and intestinal stem cell-associated pathways.

The relationship between FABP2 and the gut microbiota also warrants deeper investigation. Future studies should examine whether luminal FABP2 affects bacterial behavior, microbial community structure, short-chain fatty acid production, or mucosal immune activation. Combining microbiome sequencing, metabolomics, lipidomics, mucosal transcriptomics, spatial or single-cell analyses, and FABP2 measurement may clarify whether microbiota-targeted interventions can indirectly modify FABP2-associated pathways.

Emerging technologies may further expand the translational value of FABP2. Artificial intelligence and machine-learning models could integrate FABP2 with longitudinal clinical data, endoscopic images, histological features, microbiome profiles, metabolomic or lipidomic signatures, and other biomarkers to improve prediction of disease activity, relapse, mucosal healing, and therapeutic response. However, these applications remain exploratory and will require standardized assays, prospective validation, and external model testing before clinical implementation.

Therapeutic exploration should proceed cautiously. Rather than assuming that FABP2 is already a validated drug target, future studies should determine whether FABP2 modulation has beneficial, neutral, or harmful effects in disease-relevant models. If a causal role is demonstrated, intestinally targeted small molecules, dietary lipid modulation, or microbiota-based strategies could then be investigated as potential approaches to support epithelial homeostasis and mucosal repair.

## 5 Conclusion

FABP2 is an intestine-enriched intracellular lipid-binding protein with well-established roles in enterocyte fatty acid handling and increasing clinical interest as a biomarker of intestinal epithelial injury. In IBD, elevated circulating or fecal FABP2 levels may reflect mucosal damage, barrier disruption, and enterocyte loss, suggesting potential value for disease assessment when used alongside established clinical, endoscopic, histological, and biomarker-based tools.

Beyond its biomarker role, FABP2 has been proposed to link epithelial lipid metabolism with intestinal barrier function, microbiota-related responses, inflammatory signaling, and mucosal repair. However, many of these mechanisms remain hypothetical or are supported mainly by indirect experimental evidence, extrapolation from related lipid metabolic pathways, or studies of other FABP family members rather than direct validation in human IBD. In particular, the roles of FABP2 in intestinal stem cell regulation, PPARγ/PPARβ/δ signaling, microbiota-derived metabolites, and therapeutic modulation require further clarification.

Overall, FABP2 research in IBD remains at an early translational stage. Future studies integrating prospective human cohorts, standardized biomarker assays, intestinal organoids, experimental colitis models, microbiome and lipidomic analyses, and FABP2-specific gain- or loss-of-function approaches will be needed to determine whether FABP2 is primarily a marker of epithelial injury, an active mediator of disease biology, or a clinically useful component of multimodal disease monitoring and therapeutic stratification.
